# Health Tract, No. 232

**Published:** 1865

**Authors:** 


HEALTH TRACT, No. 232.

### SORDID.

A fine-looking old gentleman was walking along the splendid Fifth Avenue the other day, within a hundred feet of our dwelling; the gay equipages of fashion dashed by with their beautiful occupants, as if it were some gala-day, and every day is like it now; he was walking toward his princely mansion, and stooped to pick up a little rag, and turning half round, he motioned to his servant in livery, a few steps behind, to come and take it, and carry it home. If it had been a nail, or a piece of paper, or a splinter of wood, it would have been all the same, they could be used, and he could not bear to see any thing wasted; the habit of saving had become so inveterate that it was a monomania; in short, he was crazy. He may be seen any fine day, with his attendant at a respectful distance, ready at a nod to take any string or other waif that the street might furnish. His faithful wife, his elegant daughters, and his manly sons all humor him, for he was always a dear, good father and an affectionate husband when he was in his prime; they love him still for what he was, and sincerely and afflictively commiserate him for what he is. But what an occupation for a millionaire and an immortal mind to feed itself upon rags and chips, and other like mean things; to be doing this on the brink of the grave; to do it daily, when, with a different culture, that same mind might be hourly feasting on the contemplation of love divine,

"All loves excelling,"

and the hopes of soon entering upon an immortality of bliss. But the habit of saving has so grown upon him by being cherished for a lifetime, that now it is his highest happiness, his idol, and his god! The noble mind has been so tutored and so chained to the "filthy," the sordid things of time, that it can rise no higher now, but must grope and grovel on until friendly death puts out the light of life forever. What young reader can say that his life shall not have such an ending? There is only one safe, sure way of preventing the mind from making the body so completely its servant as to obey its mean, its sordid behests; and that is, not to allow the habit of saving to become so inveterate as to eat out all the nobler instincts of the higher nature. Cultivate no one passion of the mind or appetite of the body; divide your enjoyments and your indulgences, and in addition restrain them, otherwise they will gain such a power over both mind and body as to destroy both for time and eternity; not to annihilate, but to prepare them for enduring that terrible state "of blackness and darkness forever."

